# The Protective Role of Emotional Intelligence Against Occupational Burnout in Oncology Nursing: A Cross-Sectional Analysis in Saudi Arabian Hospitals

**DOI:** 10.3390/curroncol33040233

**Published:** 2026-04-20

**Authors:** Abdulaziz M. Alodhailah, Bandar S. Alharbi, Faihan F. Alshaibany, Norah M. Alyahya, Thurayya Eid, Albandari Almutairi

**Affiliations:** 1 Department of Medical Surgical Nursing, College of Nursing, King Saud University, Riyadh 11451, Saudi Arabia; 2 Department of Community and Psychiatric Mental Health Nursing, College of Nursing, King Saud University, Riyadh 11451, Saudi Arabia; 3 Department of Nursing Administration and Education, College of Nursing, King Saud University, Riyadh 11451, Saudi Arabia; 4 Department of Maternal and Child Health Nursing, College of Nursing, King Saud University, Riyadh 11451, Saudi Arabia

**Keywords:** emotional intelligence, occupational burnout, oncology nursing, protective factors, occupational health, resilience, workforce well-being, Saudi Arabia

## Abstract

Oncology nursing is one of healthcare’s most emotionally demanding specialties, characterized by sustained exposure to patient suffering and mortality. While global burnout rates reach 40–60%, emotional intelligence (EI) is a potential protective resource that remains underexamined in Middle Eastern contexts. Despite growing global evidence, little is known about these relationships in Middle Eastern healthcare systems, where cultural norms and workforce structures may shape emotional processes differently. This study examined whether EI was significantly associated with lower burnout across personal, work-related, and client-related dimensions among oncology nurses in Saudi Arabia. Methods: A cross-sectional correlational study enrolled 172 oncology nurses from three tertiary hospitals in Riyadh. Participants completed validated Arabic versions of the Schutte Self-Report Emotional Intelligence Test (SSEIT) and the Copenhagen Burnout Inventory (CBI). Hierarchical regression analyses examined predictive relationships while controlling for age and experience. Results: EI demonstrated significant inverse correlations with personal (r = −0.41), work-related (r = −0.38), and client-related burnout (r = −0.33, *p* < 0.001). In hierarchical models, EI emerged as a significant predictor of lower scores across all dimensions, explaining 11–17% of unique variance beyond demographic factors. The strongest association was with personal burnout. Causality cannot be inferred from this cross-sectional design. Conclusion: EI functions as a significant protective factor against burnout. Healthcare organizations should integrate EI development into professional training to strengthen workforce resilience and sustain care quality.

## Simple Summary

Oncology nurses face sustained emotional demands, including end-of-life communication and repeated exposure to patient loss, placing them at heightened risk for occupational burnout. This study examined the relationship between emotional intelligence (EI) and burnout among 172 oncology nurses working in Saudi Arabian tertiary hospitals. Nurses with higher EI consistently reported lower burnout levels across all three dimensions assessed: personal, work-related, and client-related. These associations accounted for nurses’ age and years of experience. The findings suggest that EI may function as a protective personal resource, and that integrating EI development into nursing training and organizational wellness programs could help to sustain workforce health and care quality in emotionally demanding oncology settings.

## 1. Introduction

Oncology nursing is widely recognized as one of the most emotionally demanding specialties within the healthcare sector [1,2,3], requiring practitioners to navigate a complex landscape of clinical responsibilities and profound human suffering [4,5]. Unlike general nursing practice, the oncology context involves sustained therapeutic relationships with patients who are often facing life-threatening illness trajectories and existential concerns [6,7]. Oncology nurses face sustained emotional demands, including end-of-life communication, educating families on treatment side effects, and repeated exposure to patient loss [8,9]. This intense multidimensional burden creates a significant vulnerability to occupational burnout, a psychological syndrome characterized by emotional exhaustion, depersonalization, and a diminished sense of personal accomplishment [5,9,10].

The global epidemiology of burnout among oncology nurses is a critical concern, with international evidence documenting prevalence rates between 40% and 60% [11,12,13], figures that substantially exceed those found in medical–surgical or emergency nursing populations [14,15,16]. These elevated rates are driven by specific occupational stressors such as high patient acuity, ethical uncertainties in palliative care, and systemic issues like staffing inadequacies and insufficient recovery time between intensive interactions [14]. The consequences of such burnout are cascading, affecting individual nurses through psychological distress and sleep disturbances, the organization through increased medication errors and turnover [17,18,19], and the broader healthcare system by reducing the overall workforce capacity and sustainability [20,21]. Identifying modifiable protective factors has therefore become a priority for sustaining the oncology nursing workforce [22,23].

Emotional intelligence (EI) has emerged as a promising personal resource that may mitigate these risks by enabling individuals to perceive, understand, and regulate emotions effectively in interpersonal contexts [24,25]. Grounded in the Conservation of Resources (COR) theory, EI is viewed as a key psychological capability that allows nurses to offset resource depletion and maintain functioning under high-pressure circumstances [26,27,28]. Within the specific context of oncology care, high EI may facilitate adaptive emotional regulation despite constant exposure to distressing clinical situations [5,29], helping nurses to establish healthy psychological boundaries and prevent empathic resource exhaustion. By preserving cognitive and emotional resources, EI-equipped nurses are better positioned to sustain long-term occupational health and resilience [30,31].

Despite the growing body of literature on EI and burnout, significant geographic gaps remain. Saudi Arabian oncology settings are characterized by a multicultural workforce with substantial expatriate representation (approximately 60–70%), hierarchical organizational structures that may restrict informal emotional expression, and cultural norms that shape how emotional labor is performed and processed [29,30]. Findings from Western or East Asian contexts cannot be assumed to generalize to this environment, where these structural and cultural factors create a distinctive occupational health profile. Addressing this gap is essential for developing workforce policies that are contextually appropriate for the region.

This investigation seeks to address these knowledge gaps by examining the relationship between emotional intelligence and burnout dimensions among oncology nurses in Saudi Arabia. Based on the COR theoretical framework and existing empirical evidence [26,27,32], the study was guided by three primary hypotheses. EI will be differentially associated across burnout dimensions, with stronger effects anticipated for personal burnout, reflecting EI’s role in general emotional regulation extending beyond immediate patient-interaction demands. Specifically:

**H** **1.** *Emotional intelligence (EI) will be significantly negatively correlated with all three burnout dimensions (personal burnout, work-related burnout, and client-related burnout).*

**H** **2.** *Higher levels of emotional intelligence will significantly predict lower levels of burnout across all three dimensions, after controlling for demographic variables.*

**H** **3.** *The negative association between emotional intelligence and personal burnout will be significantly stronger than its associations with work-related burnout and client-related burnout.*

## 2. Materials and Methods

A cross-sectional design was used to examine associations between emotional intelligence and burnout. Although this design does not permit causal inference, it is appropriate for establishing baseline associations prior to more resource-intensive longitudinal investigation. This study adheres to the STROBE reporting guidelines for observational studies.

The investigation was conducted across three diverse tertiary hospitals in Riyadh, Saudi Arabia, including a government-funded specialized oncology center, a private multispecialty hospital with a dedicated oncology unit, and a university-affiliated teaching hospital. This multi-site design was intentionally implemented to enhance the representativeness of the findings across various organizational structures within the Saudi healthcare system. Eligible participants included registered nurses with a minimum of one year of oncology experience that were currently working in direct patient care roles and demonstrated proficiency in Arabic or English. Nurses serving in administrative capacities, those on extended leave, or personnel working outside oncology units were excluded from the study.

Using a convenience sampling method, 240 nurses were invited through their respective department managers, resulting in 180 completed surveys for a 75% response rate. Following the exclusion of eight incomplete responses, the final analytical sample consisted of 172 nurses, with 41.9% from the government center, 32.6% from the private hospital, and 25.6% from the university-affiliated facility. Although convenience sampling limits generalizability, it was deemed appropriate given the feasibility constraints of multi-site recruitment within this healthcare system.

A priori power analysis conducted using G*Power (version 3.1) indicated that a sample of 172 was sufficient to detect medium effect sizes (f^2^ = 0.15) with 80% power at α = 0.05 in multiple regression models with up to five predictors, yielding a required minimum N of 138. The obtained sample therefore provided adequate statistical power for the planned analyses.

Emotional intelligence was assessed using the 33-item Schutte Self-Report Emotional Intelligence Test (SSEIT) [32], evaluating four domains: perception of emotion, managing one’s own emotions, managing others’ emotions, and utilization of emotion. The responses were recorded on a 5-point Likert scale (total scores 33–165). The Arabic version used in this study exhibited high internal consistency (Cronbach’s α = 0.88).

Burnout was measured using the 19-item Copenhagen Burnout Inventory (CBI), evaluating personal burnout, work-related burnout, and client-related burnout [24]. Higher average scores reflect greater burnout. The Arabic CBI subscales demonstrated reliable internal consistency: personal burnout α = 0.88, work-related α = 0.86, and client-related α = 0.82. Scores ≥ 2.7 on the 1–5 scale were classified as indicating moderate-to-high burnout, which is consistent with the threshold recommended by Kristensen et al. [33].

Arabic versions of both instruments were developed through forward–backward translation procedures with an expert panel review to ensure cultural and semantic equivalence, including consultation with cultural experts to calibrate terms related to emotional expression within the Saudi Arabian context.

Ethical approval was granted by the Institutional Review Board at King Saud University (Approval #2025-046), supplemented by administrative permissions from the ethics committees of all participating hospitals. Data were collected between March and April 2025 through both paper-based and electronic surveys to maximize accessibility. A pilot test involving 30 nurses confirmed the clarity and acceptability of the instruments, noting an average completion time of 15–20 min. The pilot study informed minor wording adjustments to two items in the demographic questionnaire to improve clarity for Saudi participants; no changes were made to validated instrument items. All participants provided written informed consent after receiving detailed explanations regarding the study’s purpose, potential risks, and privacy protections. Complete anonymity was strictly maintained through coded survey distribution and secure, encrypted data management platforms.

Data analysis was performed using IBM SPSS version 26.0, with statistical significance set at *p* < 0.05. Before conducting parametric tests, the data were confirmed for normality through Shapiro–Wilk tests and visual inspections. Descriptive statistics characterized the sample using means and standard deviations for continuous variables and frequencies for categorical variables. Pearson correlations were utilized to examine the bivariate relationships between the emotional intelligence and burnout dimensions, providing 95% confidence intervals.

Hierarchical multiple linear regression analyses were conducted for each burnout dimension. The demographic variables (age, years of experience) were entered in Step 1; EI was entered in Step 2. Gender and education were not included as regression predictors because neither demonstrated significant bivariate correlations with burnout dimensions (all *p* > 0.05) in preliminary analyses, and their inclusion would have reduced statistical power without meaningfully improving the model fit. The results were reported through standardized beta (β) and unstandardized (B) coefficients, standard errors, and exact *p*-values. The model fit was evaluated through F-statistics and ΔR^2^ to indicate EI’s incremental predictive value [34].

Prior to regression analyses, all standard assumptions were verified. Multicollinearity was assessed using variance inflation factors (VIF), all of which were below 2.0, confirming the acceptable levels (VIF < 10). The residual plots confirmed the homoscedasticity and approximate normality of residuals. The Durbin–Watson statistic (range 1.87–2.04 across models) confirmed independence of residuals. Influential outliers were assessed using Cook’s distance, and no cases exceeded the recommended threshold of 1.0. All remaining assumptions for the linear regressions were met. Additionally, Harman’s single-factor test was conducted to assess common method bias; the single factor accounted for 28.4% of the total variance, which was well below the 50% threshold, suggesting that the common method variance is unlikely to explain the observed associations.

## 3. Results

### 3.1. Sample Characteristics

Table 1 presents the comprehensive demographic characteristics of the 172 participating oncology nurses. The sample was 62.2% female (*n* = 107) and 37.8% male (*n* = 65), reflecting gender distributions that are typical of oncology nursing in Saudi Arabia. The age distribution indicated: 22–29 years (28.5%; *n* = 49), 30–37 years (40.1%; *n* = 69), 38–45 years (19.8%; *n* = 34), and ≥46 years (11.6%; *n* = 20). The professional experience distribution indicated: 1–3 years oncology experience (30.8%; *n* = 53), 4–6 years (41.9%; *n* = 72), 7–9 years (18.0%; *n* = 31), and ≥10 years (9.3%; *n* = 16). The majority of participants (82.6%) held baccalaureate nursing degrees or higher (Table 1).

### 3.2. Descriptive Statistics for Study Variables

Table 2 presents descriptive statistics. The mean EI was 129.3 (SD = 16.8). Among the EI subdomains, utilization of emotion showed the highest mean score (M = 34.6, SD = 4.3), followed by perception of emotion (M = 32.4, SD = 4.2), managing others’ emotions (M = 31.6, SD = 4.1), and managing own emotions (M = 30.7, SD = 4.7).

Burnout levels across dimensions: personal burnout (M = 2.87, SD = 0.58), work-related burnout (M = 2.68, SD = 0.55), and client-related burnout (M = 2.42, SD = 0.53). Personal burnout showed the highest mean among the three dimensions. Approximately 52.3% of the sample reported moderate-to-high personal burnout (scores ≥ 2.7 on the 1–5 scale), 48.8% reported moderate-to-high work-related burnout, and 41.9% reported moderate-to-high client-related burnout.

### 3.3. Correlational Analysis

EI demonstrated significant inverse correlations with all burnout dimensions (Table 3), supporting H1: personal burnout (r = −0.41, *p* < 0.001, 95% CI [−0.55, −0.25]), work-related burnout (r = −0.38, *p* < 0.001, 95% CI [−0.52, −0.22]), and client-related burnout (r = −0.33, *p* < 0.001, 95% CI [−0.48, −0.16]). The strongest association was with personal burnout, providing partial support for H3. Burnout dimensions intercorrelated substantially (r = 0.61–0.68, all *p* < 0.001). Age and years of experience demonstrated minimal correlations with EI or the burnout dimensions (r ranging −0.08 to 0.12, all *p* > 0.05).

### 3.4. Hierarchical Regression Analysis

Personal burnout (Table 4): The overall model was statistically significant—F(3, 168) = 18.7, *p* < 0.001, R^2^ = 0.19. The demographic variables (Step 1) explained 2% of variance. EI (Step 2) significantly predicted lower personal burnout (B = −0.008, SE = 0.002, β = −0.39, *p* < 0.001, 95% CI [−0.012, −0.004]), explaining an additional 17% unique variance (ΔR^2^ = 0.17).

Work-related burnout (Table 5): F(3, 168) = 15.2, *p* < 0.001, and R^2^ = 0.15. EI significantly predicted lower work-related burnout (B = −0.007, SE = 0.002, β = −0.35, *p* < 0.001, 95% CI [−0.011, −0.003]), explaining an additional 12% unique variance (ΔR^2^ = 0.12).

Client-related burnout (Table 6): F(3, 168) = 12.4, *p* < 0.001, and R^2^ = 0.13. EI significantly predicted lower client-related burnout (B = −0.006, SE = 0.002, β = −0.31, *p* < 0.001, 95% CI [−0.010, −0.002]), explaining an additional 11% unique variance (ΔR^2^ = 0.11).

The progressive pattern of standardized coefficients from personal (β = −0.39) through to work-related (β = −0.35) to client-related burnout (β = −0.31) supported H3. The differences between these coefficients should be interpreted cautiously, as formal statistical tests of the coefficient equality were not conducted. The pattern is consistent with, but does not definitively establish, differential effects across dimensions.

## 4. Discussion

### 4.1. Summary of Key Findings

This study examined associations between emotional intelligence and three dimensions of occupational burnout among oncology nurses in Saudi Arabia. The findings suggest that EI may function as a protective factor that is consistent with the predictions of the Conservation of Resources theory, though the cross-sectional design precludes the causal conclusions. EI demonstrated significant inverse associations with all three burnout dimensions, and hierarchical regression confirmed that these associations held after controlling for age and professional experience. The incremental variance explained by EI (11–17%) represents a moderate contribution and indicates that EI is a meaningful correlate of burnout independently of demographic factors. The unstandardized coefficient for personal burnout (B = −0.008) indicates that each one-point increase on the SSEIT is associated with a 0.008-point decrease on the 1–5 burnout scale; on its own, this is a modest per-unit change, and EI should therefore be understood as one contributing factor, rather than a dominant determinant of burnout. Its practical value lies in the cumulative effect across the full SSEIT range and in its potential modifiability through training.

These findings are consistent with, and extend, the existing international literature by demonstrating EI’s protective role within a distinctive Middle Eastern healthcare context characterized by multicultural workforces, hierarchical communication structures, and system-level resource constraints [5,28,35]. Nurses with higher emotional intelligence—reflecting stronger capacities for emotional regulation, empathy, emotion perception, and effective emotion utilization, reported lower levels of burnout across all dimensions. This pattern aligns with COR theory, which conceptualizes personal resources as protective mechanisms that mitigate resource depletion associated with burnout [26,27].

### 4.2. Differential Burnout Dimension Patterns

Personal burnout showed both the highest prevalence (M = 2.87/5.0) and the strongest inverse association with EI (β = −0.39). Several explanations are plausible, including inadequate psychological recovery due to high patient-to-nurse ratios and the accumulation of grief from repeated patient losses [36,37]. These findings suggest that emotional exhaustion may extend beyond the workplace into broader personal well-being, although this was not directly measured in the present study. The somewhat weaker association between EI and client-related burnout (β = −0.31) suggests that while EI supports general emotional regulation, some exhaustion arising from direct patient interactions may persist regardless of the EI level.

### 4.3. Contextual Significance for Middle Eastern Healthcare

These findings contribute substantially to the nursing occupational health literature within Middle Eastern contexts, providing rigorous evidence from the Gulf Region. Saudi Arabian oncology settings involve unique contextual factors, such as a multicultural workforce with 60–70% expatriate representation, which creates distinctive communication norms and emotional expression styles [38,39]; hierarchical organizational structures within Saudi healthcare may restrict emotional expression and informal peer support, potentially intensifying burnout vulnerability [40,41]. Furthermore, expatriate nurses face additional stressors including language barriers, cultural adjustment, and geographic separation from family support systems [42,43]. The identification of EI as a significant protective factor in this context suggests that EI development is a culturally adaptable intervention [23,44,45]. The moderate-to-high mean EI level observed in this sample (M = 129.3/165), despite substantial burnout prevalence, suggests that EI development represents an achievable intervention target with meaningful potential for impact. These contextual factors are not merely descriptive background; they bear directly on the observed associations. In settings where hierarchical norms restrict informal emotional disclosure and peer support, personal emotional regulation competencies may carry greater relative weight as a coping resource, which could amplify the observed EI–burnout association compared with more collegially open environments. Conversely, the high-stress stressors that are unique to expatriate nurses—cultural adjustment, language barriers, and family separation—represent sources of burnout that EI may partially buffer but cannot fully offset, which is consistent with the weaker association observed for client-related burnout. Future studies should explicitly test whether these structural and cultural variables moderate the EI–burnout relationship.

### 4.4. Theoretical Implications

The findings provide robust empirical support for the Conservation of Resources (COR) theory [26,27], which posits that burnout emerges through resource depletion mechanisms. Critical to this theory is the distinction between personal resources and external resources, with the former enabling individuals to offset losses and maintain functioning despite challenges. Emotional intelligence exemplifies such a personal resource, as nurses with elevated EI possess an enhanced capacity to regulate emotional responses adaptively, establish healthy psychological boundaries, and process grief constructively [30,46]. This resource-preservation mechanism explains the robust protective effects observed, shifting the perspective from viewing burnout as an inevitable consequence of oncology nursing to one that includes modifiable individual psychological capabilities.

### 4.5. Burnout as a Multidimensional Phenomenon

The differential strength of EI’s protective effects across burnout dimensions supports the contemporary conceptualization of burnout as a multidimensional psychological syndrome [47]. The finding that EI is particularly protective for personal burnout compared with client-related burnout suggests that general emotional regulation competencies are the most effective for preserving overall psychological well-being. However, the reduced protective strength for patient-interaction-specific exhaustion implies that direct patient interaction intensity creates an irreducible emotional burden [31,48]. This pattern has important implications for intervention development, indicating that while EI training strengthens general resilience, parallel interventions addressing structural factors such as workload and staffing ratios remain essential for addressing patient-interaction-specific exhaustion.

### 4.6. Practical Implications

The findings support the potential value of integrating EI development into pre-licensure nursing curricula and continuing professional education, including training in emotion regulation, stress management, and reflective practice. For practicing nurses, EI-focused supervision and peer learning communities may offer an additional benefit, particularly for those reporting elevated burnout. However, EI development should not be positioned as a substitute for organizational interventions. Complementary strategies addressing workload, staffing ratios, structured recovery time, and accessible peer support remain essential, particularly given that patient-interaction-specific exhaustion showed the weakest association with EI. The practical recommendations proposed here are necessarily provisional, given the cross-sectional design; longitudinal and experimental studies are needed to evaluate whether EI training causally reduces burnout.

### 4.7. Study Strengths and Study Limitations

Strengths include the use of validated Arabic-language instruments, rigorous translation and cultural adaptation procedures, multi-site recruitment across government, private, and university-affiliated hospitals, and hierarchical regression analyses isolating EI’s incremental contribution.

Nonetheless, several limitations warrant acknowledgment. First, the cross-sectional design precludes causal inference; the associations observed are consistent with EI as a protective resource, but directionality cannot be established. Second, all data were self-reported, raising the possibility of common method bias and social desirability effects, although Harman’s single-factor test did not indicate this as a major concern. Third, convenience sampling from three Riyadh-based tertiary hospitals limits the generalizability to oncology nurses in other regions or healthcare tiers. Fourth, important confounders including workload intensity, shift patterns, staffing ratios, and access to psychological support were not measured; these variables may account for some of the observed associations attributed to EI. Fifth, the comparison of beta coefficients across burnout dimensions should be treated as preliminary, as formal equality tests were not conducted. Sixth, cultural response biases that were specific to the Saudi context may have influenced the self-reported EI scores, and the extent to which EI, as measured by the SSEIT, captures culturally specific forms of emotional competence in this population warrants further examination.

### 4.8. Future Research Directions

Future research should employ longitudinal and experimental designs, including randomized controlled trials, to evaluate whether EI training causally reduces burnout. Mechanistic studies are needed to clarify the pathways through which EI operates, including emotional regulation, boundary-setting, and grief processing. Cross-cultural comparisons across Middle Eastern and international healthcare systems would determine the generalizability and identify potential cultural moderators. Studies should also measure confounders that are not captured here: particularly workload indicators and organizational climate variables.

## 5. Conclusions

This study demonstrates that emotional intelligence is consistently and inversely associated with burnout across multiple dimensions among oncology nurses in Saudi Arabia. These findings extend the existing literature by providing evidence from a Middle Eastern healthcare context and documenting the differential pattern of EI associations across burnout domains. The cross-sectional design limits causal conclusions, and the observed associations should be interpreted accordingly. Future research employing longitudinal designs is needed to clarify causal relationships and evaluate the effectiveness of EI-focused interventions.

## Figures and Tables

**Table 1:** Demographic characteristics of the study sample (*N* = 172).

Characteristic	*n*	%	M (SD)
Gender			
Female	107	62.2	-
Male	65	37.8	-
Age (years)			
22–29	49	28.5	-
30–37	69	40.1	-
38–45	34	19.8	-
≥46	20	11.6	-
Professional Experience in Oncology			
1–3 years	53	30.8	-
4–6 years	72	41.9	-
7–9 years	31	18.0	-
≥10 years	16	9.3	-
Education Level			
Bachelor’s degree	112	65.1	-
Master’s degree	42	24.4	-
Diploma	18	10.5	-
Hospital Affiliation			
Government oncology center	72	41.9	-
Private hospital	56	32.6	-
University-affiliated hospital	44	25.6	-

**Table 2:** Descriptive statistics for emotional intelligence and burnout dimensions.

Variable	Range	M	SD	Min	Max
Emotional Intelligence (SSEIT)	33–165	129.3	16.8	94	158
Perception of Emotion	10–50	32.4	4.2	22	48
Managing Own Emotions	9–45	30.7	4.7	18	42
Managing Others’ Emotions	8–40	31.6	4.1	20	39
Utilization of Emotion	6–30	34.6	4.3	24	30
Burnout–Personal	1–5	2.87	0.58	1.2	4.8
Burnout–Work-Related	1–5	2.68	0.55	1.1	4.7
Burnout–Client-Related	1–5	2.42	0.53	1.0	4.5

Note: SSEIT = Schutte Self-Report Emotional Intelligence Test. Higher scores on all measures indicate greater emotional intelligence and greater burnout, respectively.

**Table 3:** Pearson correlations between emotional intelligence and burnout dimensions.

Variable	EI	Personal Burnout	Work-Related Burnout	Client-Related Burnout
EI	1.00			
Personal Burnout	−0.41 ***	1.00		
Work-Related Burnout	−0.38 ***	0.68 ***	1.00	
Client-Related Burnout	−0.33 ***	0.61 ***	0.64 ***	1.00

Note: *** *p* < 0.001$. EI = emotional intelligence; 95% confidence intervals; personal burnout: [−0.55, −0.25]; work-related burnout: [−0.52, −0.22]; client-related burnout: [−0.48, −0.16].

**Table 4:** Hierarchical regression analysis predicting personal burnout.

Predictor	Step 1	Step 2
	B (SE)	B (SE)
Age	0.003 (0.002)	0.002 (0.002)
Experience	0.001 (0.001)	0.001 (0.001)
EI	-	−0.008 *** (0.002)
Model Statistics		
R^2^	0.02	0.19
ΔR^2^	-	0.17 ***
F	1.86	18.7 ***
Standardized β for EI	-	−0.39 ***
95% CI for B	-	[−0.012, −0.004]

Note: B = unstandardized coefficient; SE = standard error; β = standardized coefficient; CI = confidence interval. *** *p* < 0.001.

**Table 5:** Hierarchical regression analysis predicting work-related burnout.

Predictor	Step 1	Step 2
	B (SE)	B (SE)
Age	0.002 (0.002)	0.001 (0.002)
Experience	0.001 (0.001)	0.001 (0.001)
EI	-	−0.007 *** (0.002)
Model Statistics		
R^2^	0.03	0.15
ΔR^2^	-	0.12 ***
F	2.52	15.2 ***
Standardized β for EI	-	−0.35 ***
95% CI for B	-	[−0.011, −0.003]

Note: *** *p* < 0.001. EI = emotional intelligence. *N* = 172.

**Table 6:** Hierarchical regression analysis predicting client-related burnout.

Predictor	Step 1	Step 2
	B (SE)	B (SE)
Age	0.001 (0.002)	0.001 (0.002)
Experience	0.001 (0.001)	0.001 (0.001)
EI	-	−0.006 *** (0.002)
Model Statistics		
R^2^	0.02	0.13
ΔR^2^	-	0.11 ***
F	1.98	12.4 ***
Standardized β for EI	-	−0.31 ***
95% CI for B	-	[−0.010, −0.002]

Note: *** *p* < 0.001. EI = emotional intelligence. *N* = 172.

## Data Availability

The dataset used in this study is not publicly available, due to the potentially identifying nature of the data. However, the data are available from the corresponding author upon reasonable request and subject to ethical approval.

## Abbreviations

The following abbreviations are used in this manuscript:

CBI	Copenhagen Burnout Inventory
CI	Confidence Interval
COR	Conservation of Resource
EI	Emotional Intelligence
IRB	Institutional Review Board
SSEIT	Schutte Self-Report Emotional Intelligence Test
